# The BAFF-APRIL System in Cancer

**DOI:** 10.3390/cancers15061791

**Published:** 2023-03-16

**Authors:** Md Ashik Ullah, Fabienne Mackay

**Affiliations:** 1 Laboratory of B-Lymphocytes in Autoimmunity and Malignancies, QIMR Berghofer Medical Research Institute, Herston, QLD 4006, Australia; 2 The Department of Microbiology and Immunology, Faculty of Medicine, Dentistry and Health Sciences, School of Biomedical Sciences, University of Melbourne, Parkville, VIC 3010, Australia; 3 The Department of Immunology and Pathology, Monash University, Prahran, VIC 3004, Australia; 4 Faculty of Medicine, University of Queensland, Brisbane, QLD 4006, Australia

**Keywords:** BAFF, APRIL, BAFF-R, TACI, BCMA, B Cell, hematological cancers, solid cancers

## Abstract

**Simple Summary:**

The ligands BAFF and APRIL and their cognate receptors are critical for the differentiation, survival, and function of B cells and, as such, the maintenance of humoral immunity. The BAFF-APRIL system also modulates, either directly or indirectly, the function of other immune and non-immune cells. Clinical and experimental evidence suggests that aberrant BAFF-APRIL production impairs immune homeostasis, breaks immune tolerance, and aggravates cancer cell proliferation and invasion. Here, we have reviewed the latest understanding of the role of the BAFF-APRIL system in cancer. Greater clarity on the exact involvement of these two factors in cancer will pave the way for identifying new biomarkers for early diagnosis and developing novel therapeutic strategies.

**Abstract:**

B cell-activating factor (BAFF; also known as CD257, TNFSF13B, BLyS) and a proliferation-inducing ligand (APRIL; also known as CD256, TNFSF13) belong to the tumor necrosis factor (TNF) family. BAFF was initially discovered as a B-cell survival factor, whereas APRIL was first identified as a protein highly expressed in various cancers. These discoveries were followed by over two decades of extensive research effort, which identified overlapping signaling cascades between BAFF and APRIL, controlling immune homeostasis in health and driving pathogenesis in autoimmunity and cancer, the latter being the focus of this review. High levels of BAFF, APRIL, and their receptors have been detected in different cancers and found to be associated with disease severity and treatment response. Here, we have summarized the role of the BAFF-APRIL system in immune cell differentiation and immune tolerance and detailed its pathogenic functions in hematological and solid cancers. We also highlight the emerging therapeutics targeting the BAFF-APRIL system in different cancer types.

## 1. Introduction

BAFF and APRIL are homotrimeric type II transmembrane proteins that are proteolytically cleaved to produce soluble forms [1,2]. The soluble forms of these ligands are detected as homo- and heterotrimeric molecules, while BAFF also exists as 20-trimer assemblies under certain conditions [3,4,5]. Human BAFF and APRIL bind with high affinity to two receptors: B-cell maturation antigen (BCMA; also known as TNFRSF17) and transmembrane activator and calcium modulator and cyclophilin ligand interactor (TACI; also known as TNFRSF13B) [6,7]. There are species differences, however, as murine BAFF binds poorly to mouse BCMA [8]. TACI and BCMA can also be cleaved from the cell membrane and capture BAFF and APRIL as decoy receptors [9,10]. In addition, BAFF binds specifically to BAFF-R (TNFRSF13C) [11]. APRIL also binds to polysaccharide side chains of heparan sulfate proteoglycans (HSPGs), which does not affect the interaction of APRIL with BCMA and TACI [12]. The BAFF-APRIL system has emerged as a critical regulator of B-cell functions and associated autoimmune diseases including systemic lupus erythematosus (SLE) and blood cancers. The role of the BAFF-APRIL system in hematological and solid cancers is less well known or understood. This review aims to highlight current understanding and gaps in the knowledge.

## 2. Expression Profile of BAFF, APRIL and Receptors

BAFF mRNA is highly expressed in peripheral blood mononuclear cells (PBMCs), bone marrow (BM), and secondary lymphoid organs such as the spleen and lymph nodes, but less so in the lung, thymus, heart, placenta and small intestine [1,13]. In contrast, APRIL is detectable at low levels in healthy tissues but upregulated in many tumor cell lines [2]. In health, BAFF and APRIL are produced primarily by myeloid cells, including conventional dendritic cells (cDCs), follicular DCs, monocytes, neutrophils, and macrophages at baseline, and to a lesser extent by activated B cells [14,15]. The expression of BAFF or APRIL in these cell types increases in response to stimulation with toll-like receptor (TLR) agonists (TLR4 and TLR9), type I interferons (IFNs), IFN-γ, interleukin (IL)-10, IL-4, and transforming growth factor-β (TGF-β) in the immune synapses [14,16]. T cells are another potent source of BAFF and APRIL. While naïve T cells barely express BAFF or APRIL, in-vitro activated and differentiated T helper (Th) 1 and Th2 cells express elevated levels of BAFF and APRIL [17]. NK cells also express BAFF in a steady state, albeit at a much lower level than other immune cells [18]. However, in response to IL-2 stimulation, NK cells express significantly higher BAFF levels than monocytes [19].

Recently, cells of non-hematopoietic origin have emerged as potential sources of BAFF and APRIL. For instance, BAFF and APRIL are produced by astrocytes in the brain, especially in multiple sclerosis lesions, to support the survival of pathogenic B cells [20,21]. BAFF and APRIL are minimally expressed in primary bronchial epithelial cells at baseline, but the expression increases several-fold upon activation of the dsRNA—IFN-β pathway to support airway mucosal B-cell responses [22]. Other notable sources of BAFF and APRIL include epithelial cells in the tonsils [23] and salivary glands [24], osteoclasts [25,26], villous cytotrophoblasts and mesenchymal cells in the placenta [27], synoviocytes from rheumatoid arthritis (RA) patients [28], and breast adipocytes [29].

The observed widespread expression of BAFF and APRIL contrasts with the expression of their respective receptors, which are more restricted to specific immune cells. B cells express all the receptors of the BAFF-APRIL system, with levels of expression varying between different B-cell subsets [30]. Immature B cells express BAFF-R at a low level, which is upregulated as the B cells differentiate into more mature forms. BAFF-R upregulation is lost in terminally differentiated plasma cells (PCs) [30], and TACI expression is restricted to mature B cells and PCs [30,31]. BCMA is preferentially expressed by long-lived PCs and naïve or memory B cells [32,33,34]. Marginal zone B (MZ B) cells express higher levels of TACI compared to follicular B (Fo B) cells, and TACI expression is strongly elevated in MZ B cells upon TLR activation [35]. HSPGs have been reported to be expressed by peritoneal B1 cells [36].

Resting CD4^+^ and CD8^+^ T cells express low-level BAFF-R, which significantly increases upon TCR activation [37]. Regulatory T (Treg) cells constitutively express BAFF-R but not the other receptors [38,39]. BCMA is found to be expressed in resting and activated T-cell subsets, albeit at a much lower level than BAFF-R [37]. TACI expression in T cells is less clear. It has been detected for activated T cells [38,40] following PMA-ionomycin stimulation.

Low levels of BAFF-R expression are also observed in monocytes [41] and DCs [42] at baseline. TACI expression in myeloid cells is more dynamic. For instance, freshly isolated monocytes or monocyte-derived DCs primarily express intracellular TACI at low levels on the cell surface, which increase upon BAFF or IL-10 stimulation [41,42]. TACI is also expressed in macrophages and BM hematopoietic progenitor cells [41,43]. BCMA is expressed by monocytes and NK cells [43,44]. Plasmacytoid dendritic cells (pDCs) express the highest level of BCMA transcript among the circulating cells at baseline, with TLR7/TLR9 stimulation further upregulating BCMA expression in pDC [45,46]. Therefore, BAFF-APRIL signaling broadly impacts both innate and adaptive immunity. 

## 3. BAFF and APRIL Signaling in Immune Homeostasis

**Figure 1:**
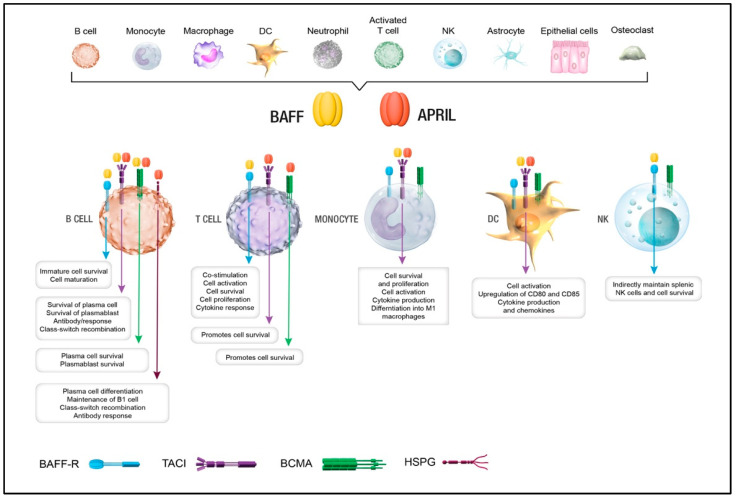
Effects of BAFF and APRIL on immune homeostasis. BAFF and APRIL are produced by immune and non-immune cells; however, the expression of the cognate receptors is restricted to specific immune cells. BAFF-APRIL binding with their corresponding receptors regulates cell differentiation, proliferation, survival, and effector functions.

BAFF-APRIL signaling regulates a diverse range of biological processes, including cell differentiation, proliferation, survival, and effector functions. Most of the reported functional studies have been conducted using clinical samples and confirmed in transgenic mouse models to ascertain the observations. Here, we summarize studies from clinical and pre-clinical models to generate a holistic picture of the BAFF-APRIL system in immune homeostasis (Figure 1).

### 3.1. Effect on B Cells

BAFF and APRIL were discovered as ligands regulating B cell differentiation, proliferation, survival, and functional responses [1,13]. BAFF and APRIL and their receptors have complementary functions in B cells. 

#### 3.1.1. B Cell Development and Differentiation

In the BM, progenitor B cells undergo a series of differentiation steps, including B-cell receptor (BCR)-mediated positive and negative selection. The immature B cells, thus formed, leave the BM and differentiate into mature B cells in the periphery [47]. BAFF-R is highly expressed by the immature B cells, and the cooperation between BAFF-R and BCR signaling facilitates the transition of immature B cells into mature B cells [48]. Survival of peripheral B cells depends on BAFF-R-mediated chromatin modifications of the NF-κB target gene promoters and upregulation of survival genes [49]. Subsequently, BAFF-R expression is downregulated while the expression of BCMA and TACI is enhanced during the differentiation of B cells to PCs [50]. Higher expression of BAFF-R and TACI has also been observed on switched memory B cells and MZB cells. Co-operation between BCR and BAFF-BAFF-R is critical for maintaining memory B cells, although contradictory results have been reported [51]. It is the precise regulation of these receptors that fine-tunes B cell differentiation.

BAFF-BAFF-R ligation is critical for mature B-cell development because mice deficient in BAFF or with inactive BAFF-R show impaired B-cell differentiation [11,52,53]. B-cell development in these mice is halted in the transitional type 1 (T1) stage and impairs the migration of the earliest, immature B cells from the bone marrow to the spleen, resulting in a deficit in mature B cells, follicular B cells, and MZB cells in the periphery [53]. A similar phenotype is also observed in mice expressing soluble TACI that neutralizes the effects of both BAFF and APRIL [8,54]. A lack of mature B cells in BAFF-deficient mice resulted in impaired humoral response to T-cell-dependent and -independent antigens [53,55]. BAFF-R-deficient mice are also immunodeficient, though less severely than BAFF-deficient mice [11] and still can induce a robust antibody response against T-cell-independent antigens, possibly via a TACI-mediated mechanism [56]. Conversely, transgenic mice overexpressing BAFF have increased numbers of mature B cells in their circulation and lymphoid organs, associated with enlarged lymphoid organs and hyperglobulinemia [57,58]. A BAFF^high^ microenvironment breaks the immune tolerance, with the survival and accumulation of autoreactive B cells, leading to the development of an autoimmune (SLE-like) disease in mice [58]. High serum BAFF levels are associated with several human autoimmune conditions [59].

APRIL appears to regulate the later stage of B-cell differentiation, primarily antigen-experienced B cells. For instance, B-cell maturation is not affected in APRIL-deficient mice [60], but APRIL is critical for the survival of long-lived PCs in the bone marrow [61]. This phenotype is more prominent in neonatal mice where APRIL expression is very low. However, the number of long-lived PCs becomes normal in aged APRIL-deficient mice, highlighting the redundant role of BAFF and APRIL in supporting long-lived PCs in BM niches [62]. In the secondary lymphoid organs, APRIL-HSPG interaction supports the splenic mature B-cell pool and differentiation into PCs [63]. Furthermore, APRIL, but not BAFF, is critical for maintaining peritoneal B1 cells, preferably via interaction with HSPGs [36].

BCMA is not critical for B-cell development and differentiation, as evidenced by a normal quantity of B cells in BCMA-deficient mice [32,53]. However, BCMA is crucial to the survival of long-lived PCs. TACI is also critical for the differentiation and survival of CD40, LPS and immunization-induced plasmablasts and PCs [64,65]. Furthermore, TACI-deficient mice exhibit mild enlargement of the spleen and lymph nodes due to the expansion of mature B cells, including follicular, MZ, and transitional B cells [66,67]. 

#### 3.1.2. B-Cell Function

BAFF and APRIL stimulate B-cell proliferation both in vitro and in vivo [1,2,13,58]. BAFF signals via BAFF-R to promote B cell survival [68]. BAFF also facilitates B-cell proliferation by increasing protein content and upregulating genes associated with glucose uptake and glycolytic metabolism [69]. The enhanced metabolic activity supports B-cell hyperactivity in response to mitogenic stimulation under inflammatory conditions [70]. BAFF also enhances mitochondrial membrane potential and attenuates oxidative stress, inhibiting cell death [71]. BAFF inhibits autophagy in healthy and neoplastic B cells by activating Akt/mTOR signaling, facilitating B-cell proliferation and survival [72]. Chronic exposure to high levels of BAFF allows self-reactive B cells to overcome metabolic restriction in the bone marrow, supporting the retention of self-reactive B cells and the production of autoantibodies, underpinning BAFF-dependent B-cell-mediated autoimmunity [70,73]. Furthermore, BAFF and APRIL induce the transcription factor AP-1 and STAT3 phosphorylation in MZ B and CD5^+^ B1 cells, causing both the differentiation and expansion of IL-10-producing regulatory B (Breg) cells under inflammatory conditions [74,75,76]. TACI signaling regulates IL-10 production by Breg cells, which suppress T-cell proliferation and cytokine production [77,78]. BAFF also induces the production of immunosuppressive IL-35 by Breg cells through the classical NF-κB pathway [79]. 

BAFF and APRIL induce the production of antibodies in response to T-cell-dependent Keyhole Limpet Hemocyanin (KLH) and T cell-independent Pneumovax antigens [68]. Mechanistically, BAFF cooperates with CD40 ligand (CD40L) by upregulating anti-apoptotic factors Bcl2/Bax, attenuating B-cell apoptosis and thus facilitating antigen-specific humoral responses [80]. BAFF also promotes survival, without enhancing proliferation, of antibody-secreting plasmablasts via a BCMA-dependent mechanism [81]. However, BAFF and APRIL have nonredundant roles in immunoglobulin (Ig) isotype class switching in B cells [16]. BAFF mediates class switching via BAFF-R and TACI, whereas APRIL induces class switching via TACI, independent of BAFF-mediated signaling [82]. Critically, heparinase treatment abrogates APRIL-mediated class-switching in B cells, suggesting that APRIL-HSPGs signaling is critical for this effect [83]. TACI and HSPG cooperate with APRIL to induce increased IgA production by B cells. The APRIL-HSPG axis also contributes to antibody responses to T-dependent antigens [63]. 

TACI regulates innate B-cell activation and maintains immune tolerance. TLR4 stimulation of MZB cells causes upregulation of TACI, followed by an increase in Fas and FasL expression and downregulation of anti-apoptotic proteins [84]. This facilitates innate activation-induced cell death (AICD) of MZB cells, to maintain immune tolerance. The ability of TACI to control immune activation and induce AICD explains the expansion of the B-cell compartment observed in TACI-deficient mice [66,67]. In mice, lack of BCMA does not affect primary and secondary antibody responses to T-cell-dependent and -independent antigens [85], because of the compensatory upregulation of TACI and subsequent B-cell activation in vivo. Thus, TACI supports antibody responses by B cells. This observation is well supported by the fact that TACI induces sustained Blimp-1 expression by B cells in response to T-cell-dependent protein antigen, (4-hydroxy-3-nitrophenyl) acetyl (NP) conjugated to ovalbumin (NP-OVA). Blimp-1 limits B-cell clonal expansion partly via inhibition of BCL-6, and promotes differentiation of long-lived antibody-secreting PCs [65]. Earlier studies reported that TACI deficiency diminishes the antibody responses to T cell-independent polysaccharide antigens but not to T-cell-dependent antigen NP-conjugated chicken γ-globulin (NP-CGG) [86,87]. These seemingly contradictory observations suggest a context/antigen-dependent role for TACI signaling in supporting PC survival and antibody production. However, such a combination of mild lymphoproliferation, autoimmunity, and immunodeficiency observed in TACI-deficient mice is also observed in individuals with mutations in the TACI protein, referred to as common variable immunodeficiency (CVID) [88,89]. However, in such a case, the dominant mutation of human TACI alone is not sufficient to drive CVID, as healthy family members of CVID patients carrying a dominant TACI mutation have been identified [90].

### 3.2. Effect on T Cells

BAFF and APRIL do not have a direct stimulatory effect on T cells. Instead, they act as co-stimulatory signals helping the activation of naïve or memory CD4^+^ and CD8^+^ T cells [68,91]. Recombinant BAFF upregulates BAFF-R and TACI expression in murine T cells [92]. However, only BAFF-BAFF-R signaling activates PI3K-Akt pathways, which promotes T-cell survival and proliferation and triggers cytokine production (IL-2, IFN-γ, IL-4, TGF-β), allowing the expansion of subsets of activated CD4^+^ T and Treg cells [91,92]. Conversely, the neutralization of BAFF-R, but not BCMA and TACI, increased the activation and cytolytic functions of CD4 and CD8 T cells [37]. These seemingly contradictory results may be attributed to the high non-physiological levels of BAFF used in one of the studies [92]. Further approaches involving the use of specific antibodies against BAFF receptors and short hairpin RNA (shRNA) gene silencing, with physiological concentrations of BAFF, specifically and perhaps more reliably addressed the relative contribution of this pathway in T-cell activation.

Interestingly, mice overexpressing APRIL in T cells do not develop splenomegaly and have normal B-cell numbers; in these mice, CD4^+^ and CD8^+^ T-cell numbers are significantly decreased [17]. However, APRIL-transgenic T cells exhibit more remarkable survival both in vitro and in vivo, possibly via the elevation of the pro-survival factor Bcl-2. These contradictory observations remain largely unexplained and require further investigation.

### 3.3. Effect on Myeloid Cells

Monocytes are primarily considered a source of BAFF. However, BAFF strongly induces monocyte survival, activation, production of proinflammatory cytokines (IL-6, TNF-α, IL-1β), and differentiation into macrophages [41]. This is further corroborated by reports that BAFF and APRIL upregulate M1 markers in macrophages and downregulate M2 markers [93]. This response is mediated by TACI signaling, as macrophages in TACI-deficient mice have an M2 phenotype and increased susceptibility to *Leishmania major* infection. BAFF also induces DC activation and upregulates costimulatory molecules CD80 and CD86, as well as the production of cytokines and chemokines [42]. This facilitates the DC-mediated proliferation of allogeneic CD4^+^ T cells and differentiation into T-helper 1 (Th1) cells.

### 3.4. Effect on Other Immune and Non-Immune Cells

The evidence of the effect of the BAFF-APRIL system on other immune cells is minimal. Pre-clinical work suggests that BAFF-BAFF-R signaling is critical for maintaining the splenic NK cell population at a steady state but is dispensable for NK cell maturation [94]. NK cells do not express BAFF receptors, neither at steady state nor in autoimmune conditions. This points towards an indirect effect of BAFF on NK cells, possible B-cell-mediated stromal cell development and follicular DC accumulation in the spleen, and production by these cells of NK-survival factor IL-15. This observation is further corroborated by previous findings that BAFF enhances the cytotoxic functions of NK cells via the upregulation of IL-2 and IFN-γ production by CD4+ T cells [95].

APRIL promotes the growth and formation of platelets via a process known as megakaryocytopoiesis [96]. Specifically, APRIL is upregulated during the growth and proliferation phase of megakaryocytic cell differentiation, and induces megakaryocyte growth. As megakaryocytes express neither BCMA nor TACI, the effect of APRIL in this axis is possibly mediated by HSPGs; a hypothesis that remains to be tested. 

Although non-immune cells express BAFF and APRIL in different tissues, non-immune cells are largely unresponsive to BAFF and/or APRIL stimulation, due to the lack of binding receptors.

## 4. BAFF-APRIL System in the Pathogenesis of Inflammatory Diseases

Dysregulation of the BAFF-APRIL system has been linked to several inflammatory conditions, specifically autoimmune diseases. Elevated levels of BAFF and APRIL have been reported in autoimmune SLE patients [59]. BAFF overexpression in mice (BAFF-Tg mice) causes accumulation of mature B cells and autoantibodies, leading to SLE [58]. BAFF facilitates the immune escape of low-affinity autoreactive B cells from negative selection during the B-cell developmental stage [13,58,73]. TLR7/9 signaling also upregulates TACI expression and TACI-dependent exaggerated B-cell response. Therefore, BAFF, in cooperation with innate immune signaling via the TLRs-MyD88 pathway, induces the production of inflammatory autoantibodies by autoreactive B cells, causing organ damage and SLE pathologies [35]. This has led to the development of the BAFF neutralizing antibody Belimumab, for the treatment of SLE and lupus nephritis [97]. 

Patients with SLE often suffer from accelerated atherosclerosis. Atherogenic and atheroprotective effects of B cells have both been reported; however, BAFF has an unusual effect on atherosclerosis. BAFF overexpression protects against hyperlipidemia and atheroma in a murine model of atherosclerosis [98], and BAFF inhibition exacerbates atherosclerosis in mice [99]. This protective response depends on B cells and BAFF-dependent TACI signaling. APRIL also limits atherosclerosis by interacting with HSPG2 in the arterial intima [100]. Hence, therapeutic inhibition of the BAFF-APRIL system in SLE patients should be carefully monitored for unwanted cardiac side effects.

Increased levels of BAFF and APRIL are also observed in other autoimmune diseases such as rheumatoid arthritis (RA), multiple sclerosis (MS), and Sjögren’s syndrome [20,21,101,102]. DCs, neutrophils, and macrophages can produce BAFF in different phases of RA progression. Excessive BAFF enhances the production of inflammatory cytokines IL-1 and IL-6 and augments the differentiation of pathogenic Th17 cells in arthritic joints [103]. BAFF also induces autoantibody production by self-reactive B cells in the joints. This inflammatory milieu causes tissue damage resulting in RA. Moreover, APRIL increases pro-inflammatory cytokine production and the accumulation of antibody-producing PCs in the joint, contributing to RA pathogenesis [104]. 

MS pathologies are primarily driven by B-cell autoantibodies, cell-mediated pathogenic T-cell differentiation, and cytokine production. However, blocking B-cell survival factors BAFF and APRIL with Atacicept exacerbates MS and increases relapse rates [105]. The protective effect of excessive BAFF in MS is further confirmed by the observation that IFN-β therapy (first-line treatment for relapsing-remitting MS) elevates BAFF levels, favoring the expansion of IL-10-producing anti-inflammatory transitional B cells instead of inflammatory class-switched memory B cells [106]. IFN-β-induced BAFF also causes the FAS-receptor/TACI-dependent apoptosis of memory B cells without affecting bone marrow PCs in MS patients [107]. BAFF overexpression in BAFF-Tg mice protects against experimental autoimmune encephalomyelitis (EAE), a pre-clinical model of MS [108]. BAFF overexpression significantly enhances the accumulation of IgA^+^ PCs/PBs and subsequent IL-10 release in the gut, in a TACI-dependent manner. The immunosuppressive milieu inhibits the production of pathogenic IFN-γ and IL-17 in the brain and ameliorates EAE pathologies. Thus, BAFF-APRIL maintains a balance of inflammatory (producing autoantibody and inflammatory cytokines) and anti-inflammatory (producing IL-10) B cells, the disruption of which can lead to MS. Further research exploring how to facilitate the BAFF-APRIL-mediated anti-inflammatory B-cell response may uncover novel therapeutic modalities for MS.

BAFF is produced in response to viral infections such as human immunodeficiency virus (HIV), respiratory syncytial virus (RSV), H1N1 influenza virus, and hepatitis C [109,110,111]. However, the induction of APRIL upon viral infection is less clearly understood. Virus-induced IFNs trigger BAFF release from myeloid cells. The augmented BAFF facilitates the expansion of HIV cross-reactive B-cell clones and atypical memory B cells in some HIV patients. Although evidence is limited of HIV-neutralizing antibodies in patients due to excessive BAFF, higher anti-HIV antibody titers can be induced with a combination of antiretroviral drugs and pegylated-IFN-α2b therapy, as demonstrated in a clinical trial [112]. Intriguingly, excessive BAFF can cause B-cell dysregulation and the development of B-cell lymphoma in HIV patients, highlighting the need to investigate further the role of BAFF in HIV infection. Furthermore, augmented BAFF signaling has been reported to maintain anti-influenza and anti-malaria antibody titers in a TACI-dependent manner [113].

## 5. BAFF and APRIL in Cancer Pathogenesis

The BAFF-APRIL system has evolved into an interconnected and complex signaling cascade that regulates cell differentiation, survival, proliferation, and migration. The BAFF-APRIL system is, therefore, a critical pathological regulator of the microenvironment, which may support cancer growth in both hematological and solid cancers.

### 5.1. Hematological Cancers

Altered expression of BAFF-APRIL and their receptors have been reported in different hematological cancers, specifically B-cell-dependent cancers (Table 1). The BAFF-APRIL system has been primarily studied in multiple myeloma (MM) and chronic lymphocytic leukemia (CLL), while recognition of its contribution to other cancers is still evolving.

#### 5.1.1. Acute Lymphoblastic Leukemia (B-ALL)

**Figure 2:**
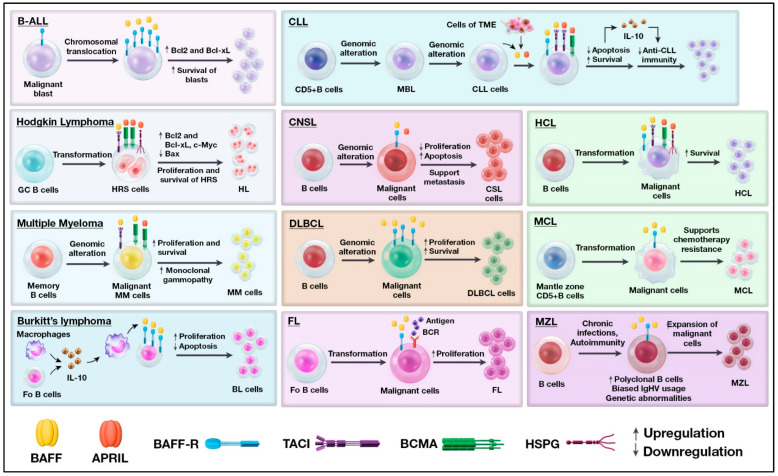
Role of the BAFF-APRIL system in the development of hematological cancers. Dysregulated BAFF-APRIL signaling primarily contributes to the proliferation and survival of cancer cells, outlined in this schematic, with the mechanisms of action described in the relevant sections. Abbreviations: B-ALL = B-cell acute lymphoblastic leukemia; GC B cells = germinal center B cells; HRS cells = Hodgkin and Reed–Sternberg cells; HL = Hodgkin’s lymphoma; PC = plasma cells; MM cells = multiple myeloma cells; Fo B cells = follicular B cells; Burkitt’s Lymphoma; CLL = chronic lymphocytic leukemia; MBL = monoclonal B-cell lymphocytosis; TME = tumor microenvironment; CNSL = central nervous system lymphoma; DLBCL = diffuse large B-cell lymphoma; FL = follicular lymphoma; HCL = hairy cell leukemia; MCL = mantle cell lymphoma; MZL = marginal zone lymphoma. ↑ refers to upregulation and ↓ refers to downregulation.

ALL can be classified into precursor B-cell lineage-ALL (Pre-B-ALL), mature B-ALL and T-cell-ALL (T-ALL) [143]. Malignant blasts appear at the pre-B-cell stage, accumulating in the BM, followed by a subsequent spread into different peripheral organs [144]. More than 50% of B-ALL patients harbor a specific chromosomal translocation t(1;19), which leads to enhanced E2A-PBX1 binding to the promoter region of the *BAFF-R* gene and increases BAFF-R expression [145]. Increased BAFF-R expression supports the BAFF-induced dual-specificity tyrosine phosphorylation-regulated kinase 1a (DYRK1a) kinase-dependent non-canonical NF-κB signaling axis in B-ALL cells, leading to the upregulation of anti-apoptotic Bcl2 and Bcl-xL, and the survival of B-ALL cells [115,146]. Thus, BAFF-BAFF-R signaling also supports the expansion of malignant B-lymphoblasts (Figure 2). This is further corroborated by the observation that DYRK1a B-cell specific–conditional knockout mice are protected from B-ALL by enhanced apoptosis and reduced proliferation of B-ALL cells. Although BAFF-BAFF-R signaling fails to inhibit apoptosis induced by crosslinking of CD24 and CD10, it protects against drug-induced apoptosis [115,116]. These findings explain the heightened level of BAFF-R expression on B-ALL cells at diagnosis, which persists and in some cases further increases during early drug treatment, suggesting the positive selection and survival of BAFF-R^high^ malignant blasts [147]. BAFF-R expression is downregulated during remission and successively increases again during relapse [147], suggesting its critical role in B-ALL pathogenesis and placing BAFF-R as a crucial therapeutic target in B-ALL.

#### 5.1.2. Hodgkin’s Lymphoma (HL)

HL develops from the clonal expansion of malignant Hodgkin and Reed-Sternberg (HRS) cells expressing CD30 [148]. Germinal center B cells transform into HRS, constituting less than 10% of the tumor mass [149]. The remaining tissue is comprised of an inflammatory infiltrate, including myeloid cells. CD30^+^ HRS cells, plasma cells, mast cells, macrophages, eosinophils and neutrophils produce BAFF and APRIL in the tumor microenvironment [33]. HRS cells lack BAFF-R but express TACI, BCMA, and HSPGs. Upon engagement with paracrine and autocrine BAFF and APRIL, these receptors upregulate intracellular pro-survival (Bcl-2, Bcl-xL) and growth-promoting (c-Myc) proteins, which signal and downregulate pro-apoptotic Bax in HRS cells (Figure 2) [33]. BAFF and APRIL also reduce the sensitivity of HRS to chemotherapeutic agents. Notably, the APRIL-BCMA axis has a more dominant effect on the proliferation and survival of HRS than the BAFF-TACI axis [33]. The dominant influence of APRIL may be attributed to HRS-anchored HSPGs facilitating the formation of APRIL oligomers, which enhance BCMA signaling in HRS. 

#### 5.1.3. Multiple Myeloma (MM)

MM is initiated by transforming the memory B cells into malignant PCs in the BM [150]. The progression of asymptomatic pre-malignant monoclonal gammopathy to active MM is driven by genomic alterations featuring somatic mutations, extra copies of chromosomes, translocations of oncogenes near the immunoglobulin enhancers, and epigenetic alterations [151]. Upon malignant transformation of PCs to active MM, the permissive microenvironment, including the BAFF-APRIL system, supports MM progression in the BM. BAFF and APRIL are produced mainly by BM stromal cells and osteoclasts, respectively [127]. BM neutrophils and monocytes also produce high levels of BAFF and APRIL. During the differentiation of long-lived PCs, BCMA expression is upregulated while BAFF-R expression decreases, and BCMA expression becomes more pronounced on the surface of MM cells [32,34,62]. Engagement of BCMA with both BAFF and APRIL supports malignant cell survival and facilitates MM progression (Figure 2) [152,153,154]. This results in excessive production of monoclonal immunoglobulins, impairing immune defenses. TACI also contributes to BAFF-mediated MM cell proliferation and survival (Figure 2) [155]. TACI is more highly expressed on Treg cells compared with conventional T cells, and APRIL-TACI interaction in Treg cells supports the immunosuppressive environment by augmenting the expression of IL-10, TGF-βa, and PD-L1, thus facilitating MM progression [156].

#### 5.1.4. Burkitt’s Lymphoma (BL)

BL originates from follicular B cells in the germinal center, including centroblasts and centrocytes [157]. The microenvironment of BL is typically characterized by significant macrophage infiltration. These macrophages and BL cells can produce high levels of IL-10 in the microenvironment [158]. In addition to its role as a classical immunosuppressant, IL-10 activates macrophages to produce BAFF (Figure 2). BAFF also induces the proliferation of BL cells [115] and inhibits the apoptosis of BL cells induced by cross-linking of BCR and CD20, thus supporting tumor growth.

#### 5.1.5. Chronic Lymphocytic Leukemia (CLL)

CLL is the most common form of adult leukemia in the aging population [159]. It is characterized by aberrant proliferation and accumulation of clonal mature CD5^+^CD19^+^ B cells in bone marrow, peripheral blood, and secondary lymphoid organs [47]. During the differentiation phase, B cells undergo oncogenic transformation induced by different chromosomal aberrations; for example, deletion of chromosome 13q (del [13q]) or acquisition of chromosome 12 (trisomy 12) [160]. Further mutations such as del [17p], del [11q], and immunoglobulin heavy-chain variable region gene (IgHV) gained over the course of the disease lead to more aggressive and treatment-resistant formof CLL and are associated with reduced survival [161,162].

Most CLL B cells are quiescent, and only a small fraction of highly proliferating cells feed the pool of leukemic cells [163,164]. Hence, impaired apoptosis and survival of leukemic cells, rather than increased proliferation, contribute to CLL cell accumulation and disease progression. The survival of leukemic cells is supported by a permissive microenvironment consisting of monocyte-derived nurse-like cells (NLCs), T cells, dendritic cells, mesenchymal stromal cells, and the cytokines and chemokines secreted by these cells [165]. BAFF and APRIL produced by CLL B cells and cells in the tumor microenvironment (TME), support CLL cell survival (Figure 2) [130,131,132,164,166]. NLCs express significantly higher levels of BAFF and APRIL in the presence of CLL cells, which is critical for CLL cell survival, as shown in ex vivo cultures [166]. Intriguingly, administration of decoy receptor BCMA (which binds to both BAFF and APRIL), but not BAFF-blocker BAFF-R:Fc, impairs the survival of CLL cells, suggesting a dominant role for APRIL in the interaction between CLL cells and NLCs. Unlike normal B cells, for which BAFF-mediated survival depend on activation of the alternative NF-κB2 pathway via BAFF-R [14], CLL cells rely on activating the classical NF-κB1 pathway via TACI and BCMA [130]. Indirect factors support the survival of CLL cells, such as immunosuppressing IL-10 which is produced by CLL cells in a BAFF-TACI-dependent manner [78]. IL-10 upregulates FoxP3 expression and subsequent Treg-mediated immunosuppression of anti-CLL immunity, facilitating CLL cell progression (Figure 2).

Despite years of research, the ligand–receptor interactions critical for CLL onset and progression in an in-vivo setting remain poorly understood. Progress has been made with the development of the Eµ.TCL1-transgenic (Tg) mouse model (TCL1-Tg model) of spontaneous progressive CLL. The T-cell leukemia -1 (TCL1) gene has been identified as an oncogene in T cell leukemia. However, elevated levels of TCL1 also have been found in human CLL and are associated with poorer prognosis. In TCL1-Tg mice, the TCL1 gene is overexpressed under the control of a VH promoter-IgH-Eµ enhancer. This targets TCL1 expression in immature and mature B cells, with the highest level observed in mature B cells [167]. The appearance of CD5^+^CD19^+^ CLL cells is observed between 8–12 months of age with the downregulation of TLR9, FLt3 expression, and decreased pDC numbers leading to impaired IFN-α production [168]. The overexpression of human APRIL in C57BL/6J mice expands CD5+CD19+ cells in the peritoneal cavity and induces a CLL-like phenotype [169]. APRIL induces B1 cell (CD5^+^CD19^+^) proliferation, which in this model was amplified in the presence of excessive APRIL. However, excessive APRIL can worsen CLL disease, as evidenced by accelerated CLL progression in APRIL-overexpressing TCL1-Tg mice [170]. This worsening of disease is driven by reduced apoptosis and increased survival of CLL cells in an APRIL-TACI-dependent manner. These observations suggest that targeting the BAFF-APRIL system in CLL may have therapeutic value. Further studies are needed to understand how this system regulates the malignant transformation of healthy B cells into CLL cells driving the disease.

#### 5.1.6. Central Nervous System Lymphoma (CNSL)

Central nervous system lymphoma (CNSL) is a rare extranodal non-Hodgkin’s Lymphoma (NHL) [171]. Most CNSL cases are of the diffuse large B-cell lymphoma (DLBCL) subtype. Primary CNSL expresses the pan-B-cell markers, including CD19, CD20, CD22, and CD79a; hence, B cells and B-cell activation signals mediated by the BAFF-APRIL system are thought to be involved in CNSL. Pathophysiologically, BAFF and APRIL do not affect tumor cell proliferation; instead, they support the chemoresistance of tumor cells by upregulating anti-apoptotic signals and boosting survival (Figure 2) [136]. BAFF and APRIL also induce chemotaxis of CNSL cell lines, supporting their role in driving tumor metastasis, as observed in CNSL patients with metastatic lung adenocarcinoma and malignant melanoma. An elegant study showed that silencing BAFF-R significantly decreases the viability and proliferation of tumor cells [172]. BAFF-R knockout tumors also exhibited delayed growth in an orthotropic mouse model of CNSL.

#### 5.1.7. Diffuse Large B-Cell Lymphoma (DLBCL)

DLBCL is an aggressive form of NHL, accounting for 30–40% of all NHL cases. A dominant role of BAFF-BAFF-R has been observed in DLBCL pathogenesis. Persistent BAFF-BAFF-R signaling promotes continuous activation of canonical and alternative NF-κB pathways, leading to autonomous proliferation and survival of lymphoma cells (Figure 2) [173]. The tumor-infiltrating neutrophils also express APRIL, which accumulates on the tumor cells via binding to HSPGs [139]. However, the pathogenic function of APRIL in DLBCL remains undetermined.

#### 5.1.8. Follicular Lymphoma (FL)

Similar to BL, FL originates from follicular B cells, centroblasts, and centrocytes in the germinal center [157]. BAFF alone is insufficient to induce FL cell proliferation in vitro, possibly due to a lower level of BAFF-R expression [133]. Co-stimulation with anti-IgM to activate the BCR is necessary to overcome the dormancy of FL (Figure 2).

#### 5.1.9. Hairy Cell Leukemia (HCL)

HCL is a rare chronic B-cell lymphoproliferative disease characterized by the high prevalence of malignant B cells with hairy morphology in the spleen and BM [174]. This results in BM failure, pancytopenia. and opportunistic infectious complications. The oncogenic pathways leading to HCL transformation remain largely unknown, including the role of the BAFF-APRIL system in this disease. HCL cells express high levels of BAFF-R, TACI, BCMA, and HSPGs and respond to BAFF and APRIL produced by splenic endothelial cells [175,176]. This results in Ig class switching and extended survival of HCL B cells (Figure 2). This function may facilitate disease progression by supporting oncogenic mutated BRAF-, BCR-, and CXCR4-mediated oncogenic pathways; however, conclusive evidence to support this hypothesis is currently lacking.

#### 5.1.10. Mantle Cell Lymphoma (MCL)

MCL is an aggressive, rare B-cell NHL that remains incurable with existing therapies. It originates from the mantle zone CD5^+^ naïve B cells [157]. Although MCL cells express both BAFF and BAFF-R, BAFF alone does not induce significant proliferation nor support the survival of MCL cells [142,177]. Instead, BAFF–BAFF-R signaling protects MCL cells from chemotherapy-cytarabine-induced cell death; thus, BAFF contributes to cytarabine resistance of MCL (Figure 2). Chemotherapy combined with BAFF-R inhibition is a possible new therapeutic strategy that may offer benefits against chemotherapy-resistant MCL.

#### 5.1.11. Marginal Zone Lymphoma (MZL)

MZL comprises three subtypes based on anatomical location: nodal MZL, extranodal MZL of mucosa-associated lymphoid tissue (MALT), and splenic MZL [178]. Chronic *Helicobacter pylori* or hepatitis C virus infection and autoimmunity support the expansion of polyclonal B cells, biased IgHV usage and genetic abnormalities of genes coding for crucial signaling molecules leading to the development of antigen-independent MZL clones. BAFF is hypothesized to support this B-cell expansion and transformation (Figure 2) [179]; however, this hypothesis remains to be tested.

### 5.2. Solid Cancers

Aberrant expression of BAFF-APRIL and its receptors has been reported in different solid cancers, as outlined in Table 2. In most cases, the BAFF-APRIL system has been found to be involved, both directly and indirectly, in tumor cell proliferation, survival, and invasion. However, the contribution of this system to tumorigenesis and metastasis remained poorly understood. In this section, we review the pathogenic functions of the BAFF-APRIL system in different solid cancers.

#### 5.2.1. Breast Cancer

**Figure 3:**
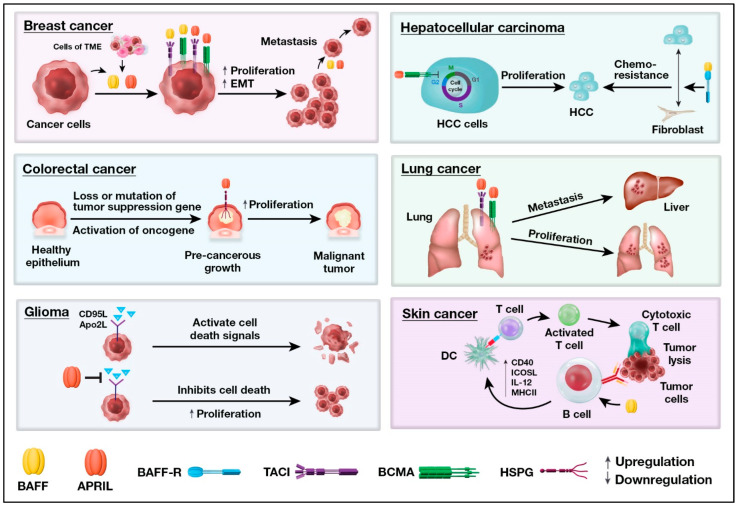
Role of the BAFF-APRIL system in the development of solid cancers. Dysregulated BAFF-APRIL signaling primarily contributes to the proliferation and survival of solid tumors, outlined in this schematic. The mechanisms of action are described in the relevant sections. Abbreviations: TME = tumor microenvironment; HCC = hepatocellular carcinoma. ↑ refers to upregulation and ↓ refers to downregulation.

APRIL is highly expressed in proliferating breast cancer cells [197]. In preclinical models, overexpression of APRIL increases the growth of 4T1 orthotopic breast tumors and promotes lung metastasis via interaction with BCMA and TACI [197]. Both BAFF and APRIL, in an autocrine or paracrine manner, can also increase the epithelial-to-mesenchymal transition and migratory capacity of malignant cells (Figure 3) [198]. These factors also upregulate pluripotency genes in cells promoting the stemness of breast cancer cells, mediated by the BCMA-JNK signaling pathway.

Interestingly, platelets express TACI and platelet-derived TACI levels are significantly elevated in breast cancer patients compared with healthy patients [182]. BAFF and APRIL do not influence platelet activation, yet activated platelets downregulate TACI expression. This is mediated by the proteolytic cleavage of surface TACI, producing sTACI that can modulate the tumor microenvironment. This is further supported by the fact that elevated TACI expression on platelets is associated with aggressive disease, incidences of metastasis, and tumor cell proliferation. In addition, silencing TACI in breast cancer cell lines halts cell division and leads to significant cell death [199]. These observations suggest a causative role of BAFF-APRIL signaling via BCMA and TACI in breast cancer growth and metastasis, indicating the BAFF-APRIL system as a potential therapeutic target.

#### 5.2.2. Colorectal Cancer (CRC)

Attenuation of APRIL expression reverses the proliferation of colon carcinoma cell line SW480. APRIL triggers cellular senescence via a mechanism that is HPSG-dependent but not TACI-dependent (Figure 3) [183]. However, the downregulation of APRIL does not induce apoptosis of tumor cells. Ectopic expression of APRIL in Apc^Min^ mice (which develop spontaneous CRC due to a mutation in the tumor suppressor gene adenomatous polyposis coli) exacerbates the abundance and size of tumors [200]. APRIL overexpression also enhances colitis-associated CRC in mice. Notably, the knockdown of APRIL in colon cancer cells inhibits both tumor clonogenicity and in vivo outgrowth, confirming the role of APRIL in CRC pathogenesis. Protection against CRC via APRIL inhibition has been confirmed using small interference RNA in subsequent studies [201,202]. However, it remains unclear how APRIL contributes to tumorigenesis and whether using an APRIL antagonist would be the right therapeutic approach in a clinical setting.

#### 5.2.3. Glioma

Gliomas are a type of invasive brain tumor with a high mortality rate. Evidence of the BAFF-APRIL system contributing to glioma pathogenesis is limited. APRIL expression in glioma cell lines is heterogeneous and does not promote glioma cell proliferation [203]. It protects cells from the cell death-inducing ligands CD95L and Apo2L but does not protect the cells from the cytotoxicity of teniposide, vincristine, lomustine, or cisplatin (Figure 3). Glioblastoma is an aggressive form of glioma. APRIL, but not BAFF, uniformly binds to most glioblastoma cell lines, and in doing so, APRIL can induce the proliferation of glioblastoma cells [185].

#### 5.2.4. Hepatocellular Carcinoma

APRIL binding to BCMA decreases HCC cell proliferation by inducing G(2)/M cell-cycle arrest and the modulation of cell-cycle-associated genes, including MCM2/4/5/6, CDC6, PCNA, and POLE2 (Figure 3) [186]. APRIL-mediated cell-cycle arrest via BCMA does not involve the classical NF-κB pathway [186]. Instead, APRIL-BCMA signaling on these cells induces a novel signaling cascade that involves JNK2 phosphorylation, subsequent FOXO3A activation, and GADD45 transcription. BAFF does not affect the growth of HCC cells. The BAFF-BAFF-R axis facilitates the interactions of HCC cells with local fibroblasts and enhances the chemoresistance of HCC [204]. Thus, HCC represents a rare system in which BAFF and APRIL exert differential effects.

#### 5.2.5. Lung Cancer

Non-small cell lung cancer (NSCLC) cell lines express BAFF and APRIL and the receptors BAFF-R and TACI, but do not express BCMA [187]. BAFF and APRIL affect the viability, proliferation, and invasiveness of NSCLC cell lines A549 and H2030. Patients with higher APRIL levels as a group has worse overall survival [205]. APRIL promotes the proliferation, migration and metastasis of NSCLC cell lines A549 and H1299 cells via BCMA and TACI (Figure 3). This contradictory result may be attributed to the different cell lines (H2030 vs. H1299) or the different generations of A549 used in the experiments. Further investigation with primary NSCLC cells is required to corroborate these findings.

#### 5.2.6. Skin Cancers

The BAFF-APRIL system has been studied primarily in skin cancer to test whether this system affects T-cell responses and promotes anti-tumor immunity. In a syngeneic mouse melanoma model (using the B16F10 cell line), BAFF upregulates CD40 and PD-L1 expression on B cells and increases CD4^+^ T cell activation, which acquires both a memory phenotype and Th1 polarization, leading to enhanced anti-tumor immunity (Figure 3) [206]. The human melanoma dataset from The Cancer Genome Atlas (TCGA) further corroborates this observation, demonstrating that elevated BAFF expression is associated with improved survival. Another study reported that tumor-adjacent keratinocytes produce high levels of APRIL in melanoma or basal cell carcinoma [180]. However, the direct effect of the BAFF-APRIL system on melanoma cell proliferation and metastasis is yet to be determined.

#### 5.2.7. Other Solid Cancers

The evidence of the pathogenic roles of the BAFF-APRIL system in the following solid cancers is limited, except for a few encouraging studies. For example, BAFF has a notable role in prostate cancer; epithelial cell-derived BAFF protects periglandular lymphocyte survival and suppresses tumor progression [207]. Hence, the lack of epithelial BAFF expression and interleukin-7 in prostate cancer facilitates tumor escape from immunosurveillance.

The association of the BAFF-APRIL system with pancreatic cancer is minimal. One study reported that BAFF and BAFF-R are primarily expressed by infiltrating B cells surrounding tumor lesions [192]. BAFF-R, but not TACI and BCMA, is also expressed by cancer cells. BAFF, via interaction with BAFF-R on cancer cells, upregulates E-cadherin, vimentin, and Snail, promoting epithelial–mesenchymal transition, tumor motility, and invasion.

BAFF expression has not been reported in cervical cancer. However, myeloid-derived suppressor cells (MDSCs) in cervical cancer patients express high levels of BAFF which, via BAFF-R, induces the expansion of IL-10-producing B cells (B10) [208]. This results in MDSC-mediated immunosuppression facilitating immune escape and progression of cervical cancer. Thus, BAFF indirectly affects cervical cancer growth.

In oral squamous cell carcinoma, tumor-associated neutrophils produced high levels of BAFF, specifically in the presence of TGF-β [190]. Neutrophil-derived BAFF binds to BAFF-R on cancer cells, promotes cancer cell proliferation, and inhibits apoptosis; thus, BAFF contributes to cancer progression.

## 6. Therapeutic Targeting of the BAFF/APRIL Pathway

BAFF-APRIL and its receptors have been causally linked to different hematological cancers. However, the evidence of their activity in solid cancers remains slim in the preliminary phase of exploration. The data for hematological cancers are more substantial, and have encouraged the development of several biologics targeting the BAFF-APRIL system to treat these cancers. In the following section, we summarize the anti-cancer drugs related to the BAFF-APRIL system that are currently progressing through different phases of drug development (Table 3). Most of these drugs are being tested in relapsed or refractory MM patients. Further drugs are in development for CLL and other blood cancers, and fewer for treating solid cancers.

### 6.1. BAFF Targeted Therapies

#### 6.1.1. Belimumab

Belimumab is an IgG1 monoclonal antibody neutralizing the soluble trimeric form of BAFF. It has been licensed to treat a subset of patients with systemic lupus erythematous or lupus nephritis [97]; its use as a cancer treatment remains under investigation. Inhibitors of BCR signaling, such as ibrutinib, idelalisib, and the Bcl-2 antagonist venetoclax are new classes of drugs for the treatment of CLL. However, resistance to these therapies has emerged as a significant problem. One of the resistance mechanisms is BAFF-mediated inhibition of apoptosis, facilitating CLL cell survival [209]. Belimumab reverses this resistance and increases the sensitivity of CLL cells to chemotherapies, independent of the clinical stage or mutation status of the CLL cells. Elevated production of BAFF by NK cells following rituximab treatment (anti-CD20 antibody) impairs NK-cell-mediated antibody-dependent cellular cytotoxicity (ADCC), causing CLL resistance [210]. This resistance to rituximab can be mitigated with the use of belimumab. Thus, repurposing belimumab to overcome drug resistance in CLL or as an adjunct therapy to small molecule inhibitors represents a promising therapeutic strategy currently being tested in clinical settings.

#### 6.1.2. Tabalumab

Tabalumab (also known as LY2127399) is a selective, fully IgG4 monoclonal antibody that neutralizes both membrane-bound and soluble BAFF. Tabalumab has been tested in phase-I and II trials in patients with relapsed or refractory multiple myeloma (RRMM), and is well-tolerated [211,212,213]. The drug, in combination with the standard regimen of dexamethasone and bortezomib, exhibited partial response (reduction in monoclonal antibody production) in approximately 50% of patients in the phase-I trials. It failed however to improve progression-free survival (PFS) in the phase-II study. One significant limitation of this combination is the prevalence of treatment-related adverse events, including thrombocytopenia, neutropenia, and pneumonia.

#### 6.1.3. BAFF Expressing CAR-T Cell

A new cell therapy using BAFF-expressing T-cells with chimeric antigen receptors (CAR-T) has been developed by Luminary Therapeutics [214]. It consists of autologous or allogeneic CD4^+^ and CD8^+^ T cells, which are genetically engineered using a non-viral transposon system to express BAFF. Approximately 30–60% of patients relapse after the use of CD19-CAR-T cells, due to the mutation of CD19 in the cancer cells. Due to the universal expression of CD19 on B cells, CD19-CAR-T cells kill both cancerous and healthy B cells. These caveats are expected to be overcome using BAFF-CAR-T cells, which target cancerous B cells but not immature B cells, expressing BAFF-R, TACI, and BCMA. BAFF-CAR-T cells have demonstrated promising efficacy in xenograft models of MCL, MM, and ALL [214]. A phase-I clinical trial has recently been initiated to treat relapsed or refractory NHL patients, primarily MCL [215].

### 6.2. APRIL Targeted Therapies

Neutralization of APRIL is an effective strategy to limit pro-survival and tumor-promoting signaling mediated by APRIL-TACI or APRIL-BCMA. An anti-APRIL neutralizing antibody has been shown to inhibit myeloma cell propagation and limit cell adhesion and migration in vitro [153,216]. The anti-APRIL blocking antibody also exerts a cytotoxic effect and suppressed human MM growth in a humanized mouse model [153]. APRIL-based chimeric antigen receptor (CAR) T cells, which recognize both BCMA and TACI, have been developed and found to be effective in killing MM cells and providing tumor regression, even at very low BCMA and TACI densities [217]. Further improvements with trimeric APRIL CAR-T cells showed greater efficacy in limiting MM growth [218]. These recent discoveries are now being evaluated in phase-I clinical trials.

### 6.3. BAFF-R Targeted Therapies

BAFF-R targeting antibody VAY-736 is a humanized defucosylated IgG1 monoclonal antibody. VAY-736 alone has shown superior antibody-dependent cellular cytotoxicity against CLL, compared with anti-CD20 and anti-CD52 antibodies. It also prolongs the survival of CLL mice when given in combination with ibrutinib (a Bruton tyrosine kinase inhibitor used to treat CLL), compared with either treatment alone [219]. BAFF-BAFF-R-mediated activation of non-canonical NF-κB signaling remains elevated despite ibrutinib treatment; hence, the combination of ibrutinib and VAY-736 exerts a synergistic response against CLL. VAY-736 also facilitates NK-cell-mediated killing of MCL cells in vitro and in vivo [177]. In addition, VAY-736 enhances NK-cell-mediated antibody-dependent cellular cytotoxicity to eliminate advance-stage drug-resistant B-ALL cells [220]. A synergistic anti-cancer response is obtained when VAY-736 is combined with TGF-β1 inhibition, overcoming TGF-β1-mediated suppression of NK cell function. These observations have prompted several phase-I studies of VAY-736 in NHLs. Recently developed BAFF-R-targeted and CD19/BAFF-R-targeted CAR-T cells have also been shown to promote durable remissions in B-ALL and MCL [221,222]. These are currently being tested in phase-I studies with relapsed or refractory ALL and MCL patients.

### 6.4. BCMA Target Therapies

Therapies targeting BCMA have primarily been developed to treat MM, due to its robust and highly specific expression on MM cells. One of the major concerns in developing BCMA-targeting biologics is that BCMA on the cell surface is cleaved by γ-secretase, resulting in the downregulation of cell-surface BCMA [10] and limiting the efficacy of BCMA-targeted biological drugs on cancer cells. This has encouraged the development of dual-targeted therapies, including bispecific antibodies (bsAbs), antibody-drug conjugates (ADCs), and CAR-T cells. These therapies are at varying stages of preclinical and clinical development.

#### 6.4.1. Bi-Specific Antibodies

Bi-specific antibodies bind to malignant MM cells using an anti-BCMA single-chain variable fragment (scFv), and activate cytotoxic T cells via the other arm containing anti-CD3ε scFV [223]. This bi-specific antibody strategy facilitates cytotoxic T-cell-mediated killing of MM cells in an MHC-independent manner, bypassing the complex interaction between cancer antigen, dendritic cells and T cells. AMG420 (also known as BI 836909) is a first-in-class, bispecific T-cell engager (BiTE^®^; Thousand Oaks, CA, USA) [224]. AMG420 induces selective lysis of BCMA-positive MM cells with potent T-cell activation and cytokine secretion [225]. In phase-I clinical trials, AMG420 demonstrated a 70% response rate in relapsed or refractory MM (RRMM) patients, with 50% of responders achieving minimal residual disease. However, AMG420 has a shorter half-life and a new generation of antibodies with an increased half-life is currently under development. For instance, EM801 is constructed as an asymmetric two-arm IgG1 humanized antibody with a modified Fc region, to prolong its half-life [226]. One arm exhibits divalent binding with BCMA, while the other has a weaker affinity for CD3ε on T cells, reducing the toxicity driven by excessive activation of T cells. Yet, EM801 induces superior cross-linking between CD3^+^ T and MM cells, activates CD4^+^ and CD8^+^ T cells, and causes secretion of interferon-γ, granzyme B, and perforin to kill MM cells. In the phase-I clinical trial, EM801 demonstrated promising efficacy, with more than 50% of RRMM patients experiencing partial or better response [227]. However, EM801-driven side effects, remain significantly limiting, including thrombocytopenia, neutropenia, anemia, infections, and cytokine release syndrome. AMG701 is another anti-BCMA BiTE with a long half-life (112 h) and robust functionality, undergoing clinical trials in RRMM patients [228]. Teclistamab has also shown promising efficacy in RRM patients, with 78% of patients exhibiting partial or better response [229,230]. A few other candidates, PF-06863135, REGN-5458, REGN-5459, and TNB-383B are currently progressing through different phases of clinical trials in MM [231].

#### 6.4.2. Antibody-Drug Conjugates (ADC)

ADCs are complex molecules composed of three components: (i) a tumor-associated antigen (TAA)-targeting monoclonal antibody; (ii) a cytotoxic drug, e.g., the tubulin polymerization inhibitors monomethyl auristatin F (MMAF) or monomethyl auristatin E (MMAE), the DNA double-strand breaking agent calicheamicin, pyrrolobenzodiazepine (PBD), which are DNA minor-groove crosslinking agents, or other toxins; (iii) a linker that joins the two components [232]. When the antibody binds to its target cell, ADC is internalized, and the cytotoxic drug is released to induce cell damage and death.

Belantamab Mafodotin (GSK2857916) is the first anti-BCMA ADC undergoing clinical trials. It is an afucosylated, humanized IgG1 monoclonal antibody conjugated with MMAF through a non-cleavable maleimidocaproyl linker [46]. Belantamab Mafodotin displays enhanced killing and decreased toxicity through three known mechanisms. The first is the ADCC effect, mediated by NK cells, which is increased by defucosylation of the constant region carbohydrate fragments. Secondly, after lysosomal endocytosis, the release of the toxin MMAF in the cytoplasm inhibits microtubule polymerization and induces apoptosis. Lastly, the non-cleavable linker prolongs the stability of the ADC in the blood, with limited toxicity toward off-target cells [46]. Belantamab Mafodotin has shown promising MM regression (up to 3.5 months) in a xenograft mouse model. This ADC has shown favorable responses in various clinical trials with an overall response rate of ~30% [233]. Recently, FDA has approved the drug for treating adult RRMM, under a risk evaluation and mitigation strategy with a warning of ocular toxicity. Several other ADCs, including MEDI2228 (human anti-BCMA antibody conjugated to a pyrrolobenzodiazepine dimer via a protease-cleavable linker) and HDP-101 (anti-BCMA antibody conjugated to amantin toxin), have shown promising efficacy in preclinical models [234,235] and are currently in phase-I clinical trials.

#### 6.4.3. BCMA Expressing CAR-T Cells

Several BCMA-directed CAR-T cell therapies have been developed with promising therapeutic efficacy in recent years. CAR-T cells targeting BCMA/CD28/CD3ζ exhibited a potent anti-myeloma response in two clinical trials with heavily treated RRMM patients [236,237]. Later work revealed that CD137 co-stimulation was superior to CD28 co-stimulation, and a new CAR-T cell was developed: Idecabtagene vicleucel (bb2121), which comprises scFv specific to BCMA, attached to a human CD8 α hinge, a CD137 costimulatory motif, and CD3ζ signaling domain [238]. Bb2121 has a promising anti-myeloma effect, with 45% to 73% of patients exhibiting partial or better response and 26% of patients achieving minimal residual disease, with negative status for up to one year [238,239]. FDA has recently approved Bb2121 as the first CAR-T cell for RRMM treatment. A modified version of bb2121, LCAR-B38M CAR-T targeting two BCMA epitopes, has also been developed and shown an 88% overall response, with 63% of patients achieving minimal residual disease and negative status [240].

Despite the revolutionary success, loss of surface BCMA antigen on MM cells, expansion of BCMA-negative clones, T-cell dysfunction, and the immunosuppressive milieu contribute to relapses, which remain a major concern [241,242]. This has encouraged the exploration of other myeloma antigens, such as CAR-T cells targeting BCMA/TACI, BCMA/CD19, and BCMA/CD38, which are currently in clinical trials [243,244,245]. Each of the CAR-T cells currently in clinical trials has significant adverse effects, such as leukopenia, thrombocytopenia, liver toxicity, and cytokine release syndrome. Hence, the development of CAR-T cells with more robust functionality, efficacy, and reduced toxicity, is needed and is in progress.

#### 6.4.4. BCMA Decoy Receptor

Soluble BCMA decoy receptor (sBCMA-Fc) offers a superior response compared with the BCMA-specific therapies mentioned above. sBCMA-Fc binds to BAFF and APRIL with high affinity, and thus overcomes failure of treatment caused by the downregulation of cell-surface BCMA. Miao and colleagues have recently developed an affinity-enhanced sBCMA-Fc fusion protein which has shown promising antitumor activity in both MM and DLBCL [246]. Notably, the fusion protein has demonstrated a favorable safety profile and target-specificity in non-human primates, bypassing the drug-related toxicities (cytokine storm, tissue damage, neuropathy, and ocular toxicity) of bi-specific antibodies, ADCs, and CAR-T cells. This makes sBCMA-Fc a promising therapy for vulnerable patients, however, its efficacy is yet to be thoroughly tested in the clinic.

### 6.5. TACI Target Therapies

BAFF and APRIL blocking for cancer treatment has been achieved using decoy fusion proteins. Atacicept is a soluble decoy fusion protein composed of the extracellular domain of the TACI receptor fused with the FC domain of human IgG1. Atacicept blocks soluble and membrane-bound BAFF and APRIL, inducing the apoptosis of MM cells and reducing myeloma burden in humanized mice [247]. Atacicept has demonstrated promising results in a phase I clinical trial, with most RRMM patients reporting progression-free status [248]. Significantly, atacicept decreases the polyclonal Ig isotypes and circulating Ig-producing B cells. In another phase-1b clinical trial, Atacicept demonstrated a moderate effect in fludarabine-refractory CLL patients, with approximately 45% of patients attaining disease stability [249]. The lack of a robust response to atacicept in MM and CLL has halted further clinical trials. The only TACI-targeting CAR-T cell currently under development is a bispecific TACI/BCMA CAR-T cell which has shown a potent cytotoxic response towards MM cells both in vitro and in vivo [245].

## 7. Conclusions and Perspective

The BAFF-APRIL system is a vital feature of the immune system, essential to maintaining B cells and humoral immunity. The BAFF-APRIL system plays an instrumental role in inflammatory reactions underlying chronic inflammatory diseases, including autoimmunity. In this review, we have gathered a large body of evidence demonstrating the role of this system in hematological and solid cancers. The expression of BAFF, APRIL and/or their respective receptors is often considered a biomarker of cancer progression and responses to therapy. However, the role of the BAFF-APRIL system in the initial malignant transformation and progression of cancers remains largely unexplored, especially in solid and non-B-cell hematological cancers. The BAFF-APRIL system has been well exploited to develop a wide range of new therapeutics against hematological cancers, particularly MM. While these new products are yet to be validated in the clinic, and with some being associated with significant toxicity, the growing focus on the refinement of therapies targeting the BAFF-APRIL system is a testament to the crucial importance of this system in cancer progression. One of the major challenges in developing therapeutics is targeting the BAFF-APRIL system with efficacy while maintaining protective B cell-dependent immunity and reducing associated side effects. The COVID-19 pandemic and vital vaccination programs have brought this consideration center-stage for the development of future treatments. Much less is known about the role of the BAFF-APRIL system in solid tumors. In this review we have detailed some interesting results, but the limitation of most existing studies is the lack of clear distinction between the role of BAFF or APRIL on tumor cells as opposed to tumor-infiltrating immune cells. Future work should take advantage of novel techniques such as single-cell sequencing and spatial transcriptomics, to better outline immune-related and tumor-related mechanisms of cancer progression controlled by the BAFF-APRIL system.

## Figures and Tables

**Table 1:** Expression profile of the BAFF and APRIL system and clinical relevance in hematological cancers.

Hematological Cancers	Expression Profile and Clinical Relevancy
B-ALL	Serum BAFF and APRIL concentrations are higher in B-ALL patients [114]. BAFF-R expression in primary B-ALL cases is heterogeneous (0.9% to 93% of B-ALL cells express BAFF-R) [115]. TACI and BCMA expression is relatively low to negative in B-ALL cells [115,116].
HL	Increased serum levels of BAFF and APRIL in HL [117].
MM	Serum levels BAFF and APRIL are three to five times higher in MM [118,119]. Higher BAFF expression is associated with disease progression and shorter progression-free survival [118,120,121,122]. BAFF-R expression is very low or absent in primary MM cells and in MM cell lines [119]. BCMA is highly expressed in the plasma cells of MM patients, and serum soluble BCMA (sBCMA) levels are used as a biomarker for MM disease status [123,124]. Patients with higher levels (above 326.4 ng/mL) of sBCMA have significantly shorter progression-free (3.6 months) and overall survival (98 months) than patients with less than 326.4 ng/mL of serum sBCMA (9.0 and 155 months, respectively) [125]. Higher TACI expression is observed in on MM cells [126,127]; however, lower TACI expression is associated with worse prognosis, including increased stage III MM probability, attenuated hemoglobin levels, and increased bone lesions [127].
NHL	
BL	BAFF-R expression in primary BL cases is heterogeneous (0.04 to 81% of B-ALL cells express BAFF-R) [115].
CLL	Increased BAFF level in CLL patients, specifically with unmutated IgHV, and increased BAFF expression is associated with worse outcomes [128,129]. The plasma level of APRIL is higher in CLL patients [128,129]. Higher intracellular APRIL and BAFF in CLL cells is associated with higher expression of adverse prognostic factors CD38 and ZAP70 and poorer clinical outcomes [128]. The expression of BAFF-R, TACI and BCMA on CLL B cells is comparable to healthy B cells [130,131,132]; however, lower BAFF-R expression on CLL B cells has also been reported [133]. CLL B cells with mutated IgHV express more TACI and BCMA than unmutated cells [132]. Plasma sBCMA levels are significantly higher in CLL patients, with the sBCMA concentration increasing with disease severity and associated with poorer outcomes [134,135].
CNSL	Elevated levels of BAFF-APRIL and their receptors BCMA and TACI have been detected in the cerebrospinal fluid (CSF) and biopsies of CNSL patients, compared to patients with other neurological diseases [136,137,138]. The levels of BAFF and APRIL in CSF act as sensitive and specific biomarkers for CNSL diagnosis and therapeutic response. The serum levels of BAFF or APRIL remain unchanged, suggesting a localized response.
DLBCL	BAFF-R expression is comparable to healthy B cells [133]. Tumor cells also express TACI, BCMA and HSPGs [139]. Serum BAFF concentrations and APRIL expression in tumor lesions are higher and associated with poor prognosis [139,140].
FL	Lower BAFF-R expression [133]. Three-fold higher serum BAFF expression compared to healthy donors [133].
HCL	HCL cells express high levels of BAFF-R, TACI, BCMA, and HSPGs [141].
MCL	Serum BAFF concentrations are higher and correlate to poor treatment response and relapse [142]. BAFF-R expression is comparable to healthy B cells [133].
MZL	BAFF-R expression is comparable to healthy B cells [133].

Abbreviations: B-ALL = B-cell acute lymphoblastic leukemia; BCP = B-cell precursors; HL = Hodgkin’s lymphoma; MM = multiple myeloma; NHL = non-Hodgkin’s lymphoma; BL = Burkitt’s lymphoma; CLL = chronic lymphocytic leukemia; CNSL = central nervous system lymphoma; DLBCL = diffuse large B-cell lymphoma; FL = follicular lymphoma; HCL = hairy cell leukemia; MCL = mantle cell lymphoma; MZL = marginal zone lymphomas.

**Table 2:** Expression profile of BAFF and APRIL system in solid cancers.

Solid Cancers	Expression Profile and Clinical Relevancy
Breast cancer	BAFF is ubiquitously expressed in malignant and non-malignant tissue, whereas APRIL expression is lower in malignant tissues than in non-malignant tissues [29]. Other studies reported overexpression of APRIL protein and mRNA in tissue lesions [180,181]. None of the receptors can be identified immunohistochemically, yet the mRNAs of the receptors are detectable, most likely related to tumor-infiltrating immune cells [29,182].
Colorectal cancers	Higher levels of APRIL mRNA have been reported [2]. Colorectal cancer cell lines express higher levels of HSPG, with only marginal expression of BCMA and TACI observed [183].
Glioma	Higher levels of APRIL, BCMA, and TACI expression and lower BAFF protein levels in glioma [184]. BAFF, APRIL, and their receptors are expressed by the majority of glioblastoma (a type of glioma) cell lines [185].
HCC	APRIL and BCMA levels are higher, whereas BAFF and BAFF-R expressions are unchanged in HCC [186]. HCC does not express TACI.
Lung cancer	The non-small cell lung cancer (NSCLC) cell lines express BAFF and APRIL and the receptors BAFF-R and TACI, but do not express BCMA [187].
Neuroendocrine tumors	Higher BAFF expression is associated with disease severity and refractory disease [188,189].
Oral squamous cell carcinoma	Overexpression of BAFF and APRIL [190,191].
Pancreatic cancer	Serum BAFF concentration is significantly higher and associated with disease severity and metastasis [192].
Renal carcinoma	BAFF, APRIL, and TACI are highly expressed in cancer biopsies, while the expression of BAFF-R and BCMA is very low to undetectable [193]. Higher levels of APRIL expression in biopsies are associated with disease severity and negatively correlated with disease-free survival.
Skin cancer	Higher expression of BAFF, APRIL, and BCMA in skin cancers, specifically in uveal melanoma, has been reported [194,195]. High BAFF expression is a predictor of metastasis.
Thyroid carcinoma	Higher APRIL mRNA expression is found in thyroid carcinoma [2].
Others	Serum concentrations of BAFF in nephroblastoma (Wilms’ tumor), Ewing sarcoma, and rhabdomyosarcoma were higher than in healthy controls and the childhood non-Hodgkin’s lymphoma subgroup [196].

Abbreviations: HCC = hepatocellular carcinoma.

**Table 3:** Drugs targeting the BAFF-APRIL system.

Target	Drug Name	Developmental Phase	Indication
Anti-BAFF antibody	Belimumab	Phase II	Relapsed and/or refractory CLL
	Tabalumab	Phase II	Relapsed and/or refractory MM
BAFF CAR-T cells	LMY-920	Phase I	Relapsed and/or refractory NHL
Anti-APRIL antibody	BION-1301	Phase I	Relapsed and/or refractory MM
APRIL CAR-T cells	APRIL CAR-T	Phase I	Relapsed and/or refractory MM
	AUTO2	Phase I/II	Relapsed and/or refractory MM
Anti-BAFF-R antibody	VAY-736	Phase I/Ib	NHLs (DLBCL, FL, MCL, MZL)
BAFF-R CAR-T cells	BAFF-R CAR-T	Phase I	Relapsed or refractory B-cell ALL and MCL
Anti-BCMA and anti-CD3ε bispecific antibody	AMG420	Phase I	Relapsed and/or refractory MM
	AMG701	Phase I/II	Relapsed and/or refractory MM
	Teclistamab	Phase II/III	Relapsed and/or refractory MM
	Elranatamab	Phase II/III	MM
	REGN-5458	Phase I/II	Relapsed and/or refractory MM
	TNB-383B	Phase I	Relapsed and/or refractory MM
Anti-BCMA ADC	Belantamab Mafodotin	Phase III/FDA approved	Relapsed and/or refractory MM who has received at least four prior therapies
	MEDI2228	Phase I	Relapsed and/or refractory MM
	HDP-101	Phase I/II	Relapsed and/or refractory MM
BCMA CAR-T cells	Idecabtagene vicleucel	Phase II/FDA approved	Relapsed and/or refractory MM and MM patients received hematopoietic stem cell transplantation
	LCAR-B38M CAR-T	Phase II	Relapsed and/or refractory MM
	BCMA/CD19	Phase I	Relapsed and/or refractory MM, B-ALL
	BCMA/CD38	Phase I/II	Relapsed and/or refractory MM
TACI-Fc-chimera protein	Atacicept	Phase I	Relapsed and/or refractory MM and CLL

Abbreviations: ADC = antibody–drug conjugates; CAR-T = T cells with chimeric antigen receptors. Source: ClinicalTrials.gov database.
